# A protein-based set of reference markers for liver tissues and hepatocellular carcinoma

**DOI:** 10.1186/1471-2407-9-309

**Published:** 2009-09-02

**Authors:** Stella Sun, Xin Yi, Ronnie TP Poon, Chun Yeung, Philip JR Day, John M Luk

**Affiliations:** 1 Department of Surgery, LKS Faculty of Medicine, Jockey Club Clinical Research Centre, The University of Hong Kong, Pokfulam, Hong Kong; 2 Quantitative Molecular Medicine, The Manchester Interdisciplinary Biocentre, University of Manchester, Manchester, UK; 3 Departments of Pharmacology and Surgery, Yong Loo Lin School of Medicine, National University of Singapore, Singapore

## Abstract

**Background:**

During the last decade, investigations have focused on revealing genes or proteins that are involved in HCC carcinogenesis using either genetic or proteomic techniques. However, these studies are overshadowed by a lack of good internal reference standards. The need to identify "housekeeping" markers, whose expression is stable in various experimental and clinical conditions, is therefore of the utmost clinical relevance in quantitative studies. This is the first study employed 2-DE analysis to screen for potential reference markers and aims to correlate the abundance of these proteins with their level of transcript expression.

**Methods:**

A Chinese cohort of 224 liver tissues samples (105 cancerous, 103 non-tumourous cirrhotic, and 16 normal) was profiled using 2-DE analysis. Expression of the potential reference markers was confirmed by western blot, immunohistochemistry and real-time quantitative PCR. geNorm algorithm was employed for gene stability measure of the identified reference markers.

**Results:**

The expression levels of three protein markers beta-actin (ACTB), heat shock protein 60 (HSP60), and protein disulphide isomerase (PDI) were found to be stable using p-values (*p *> 0.99) as a ranking tool in all 224 human liver tissues examined by 2-DE analysis. Of high importance, ACTB and HSP 60 were successfully validated at both protein and mRNA levels in human hepatic tissues by western blot, immunohistochemistry and real-time quantitative PCR. In addition, no significant correlation of these markers with any clinicopathological features of HCC and cirrhosis was found. Gene stability measure of these two markers with other conventionally applied housekeeping genes was assessed by the geNorm algorithm, which ranked *ACTB *and *HSP60 *as the most stable genes among this cohort of clinical samples.

**Conclusion:**

Our findings identified 2 reference markers that exhibited stable expression across human liver tissues with different conditions thus should be regarded as reliable reference moieties for normalisation of gene and protein expression in clinical research employing human hepatic tissues.

## Background

Quantitative proteomic and genomic technologies have recently revolutionized the search for disease-specific biomarkers or molecular signatures that allow early prognosis and accurate detection of illness. Functional studies of these gene and protein biomarkers also facilitate our better understanding of pathogenesis pathways in relation to disease onset and progression (e.g. carcinogenesis and tumour metastasis) [1-3]. However, we often encounter the unwanted scenario that associates biological variability in clinical specimens with respect to the corresponding disease phenotypes and tissue qualities at the time of collection [4,5]. These intrinsic factors significantly hinder data analysis and accurate interpretation in genome- and proteome-wide expression profiling studies [2]. To better decipher biological and experimental variations, the inclusion of certain specifically selected internal reference control(s) for data standardization or normalization can facilitate accurate biomarker comparisons.

Ideally, a "good" internal reference marker is expected to show a constant level of expression presence across all tissue samples of the same type and of the same experimental design and treatment. Nevertheless, there is mounting evidence that the conventional transcripts or proteins used as ubiquitous internal house-keeping controls such as glyceraldehyde-3-phosphate dehydrogenase (GAPDH), beta-actin and beta-tubulin, are often variable in expression levels across different sample types and experimental conditions [6-8]. In this regard, there are unmet needs to identify and validate a set of stable reference markers for data standardization in human liver tissues.

The availability of protein reference markers for quantitative comparison is perhaps more enigmatic than transcriptional counterparts. This is largely contributed by differential extraction and binding efficiencies affecting measurement of proteins, plus analytical measurement anomalies which are largely absent with transcript measurement. To date, the expression stabilities of conventional reference protein markers have not yet been examined systemically in human liver tissues and during the pathogenic course of tissue transformation from the healthy or preneoplastic (cirrhosis) conditions to the cancerous stage (hepatocellular carcinoma, HCC) [8]. This inevitably affects an accurate prediction of the protein expression profiles among different clinical samples, particularly when data normalization is based on the internal controls that are considered likely to be sub-optimal in their expression stabilities. To address this deficiency, we employed the 2-DE based proteomic approach to search for potential reference protein markers whose expression levels are evenly present in clinical specimens obtained from HCC tumours, cirrhosis and healthy livers, hereafter confirmed by quantitative approaches. In fact, the general correlation between protein and transcript expression remains unclear and without consensus, the candidate reference markers were further subjected to cross-examination together with other conventional reference gene markers by employing the geNorm algorithm in order to determine the corresponding gene expression stabilities.

## Methods

### Clinical samples

A total of 224 resected clinical liver samples (105 tumours, 103 non-tumourous cirrhotic and 16 normal) were collected from patients with informed consent after surgical operation at the Department of Surgery, Queen Mary Hospital (Pokfulam, Hong Kong) between 1998 and 2005.

### Two-dimensional gel electrophoresis (2-DE) and mass spectrometry (MS)

Sample preparation and the 2DE procedure were performed as previously described [9]. In brief, proteins were extracted from 20 mg liver tissue samples using the ReadyPrep™ Sequential Extraction Kit (Bio-Rad, Hercules, CA, USA) and protein concentration of the lysates was quantified using the PlusOne 2-DE Quant Kit (GE Biosciences, Buckinghamshire, England). Isoelectric focusing was carried out using a 30 μg aliquot of protein lysate placed on a 11 cm strip (pH 4-7) (GE Biosciences), with running conditions performed as described [9]. For the second dimension gel electrophoresis, the proteins were resolved on 1 mm thick 12.5% precast gels that were separated overnight under a constant current of 10 mA per gel in an Ettan™ DALTsix Electrophoresis Unit (GE Biosciences) at 10°C. After gel fixation and silver staining (GE Biosciences), the protein expression patterns resolved on the gels were captured with a GS-800 Calibrated Densitometer (Bio-Rad) and the images were analysed using PDQuest version 8.0 (Bio-Rad). The intensity of each spot was normalised by total valid spot volume and was reported as a relative value in ppm.

The selected protein spots were digested in gel according to the method described by Yi, X *et al *[10] using MS grade trypsin (Promega, Madison, WI, USA). The peptide masses were determined using a MALDI-TOF mass spectrometer. Full scan and product ion-mass spectra were acquired on a hybrid quadrupole-time of flight mass spectrometer (QSTAR-XL™ or Voyager-DE™ STR, Applied Biosystems Inc., Foster City, CA). The tandem mass spectra were collected in product ion mode for the peptides of interest. Measured peptide and fragment ion masses were used for protein identification by two online programs: MS-FIT http://prospector.ucsf.edu and MASCOT http://www.matrixscience.com.

### Western Blot analysis

Verification of protein content and quantity was carried out in a new set of 20 liver tissues which included 4 cirrhosis, 4 paired tumour and peri-tumour of less than 2 cm; 4 paired tumour and peri-tumour of greater than 2 cm. Protein extracts were prepared as previously described [10]. Extracted protein lysates were subjected to 10% SDS-PAGE electrophoreses and transferred to a nitrocellulose membrane. Tris-buffered saline (TBS) containing 5% non-fat milk was used for blocking 1 h at room temperature before incubation with primary rabbit polyclonal anti-HSP 60 (1:1000 dilutions) and mouse monoclonal anti-beta-actin antibodies (1:1000 dilutions) (Cell signalling technology, Danvers, MA, USA and Sigma, St. Louis, MO, USA respectively). The blots were washed and incubated with horseradish peroxidise-conjugated secondary antibodies (1:10,000 dilutions) followed by the development with ECL detection system (GE Biosciences). Densitometry data were analysed by Quantity One (Bio-Rad) for semi-quantification of HSP 60 and beta-actin.

### Immunohistochemistry

To further confirm the results from the 2-DE, immunohistochemical analyses on pathologist reviewed tissue sections were executed as previously described [11]. Endogenous peroxidase activities of the sections were quenched with 3% hydrogen peroxide and the sections were blocked using 1% bovine serum albumin (BSA) (w/v in PBS). Antibodies against β-actin (Sigma) and HSP60 (Stressgen Biotechnologies, Victoria, Canada) were added separately to the tissue sections, which were then incubated at 4°C overnight. Thereafter, the sections were incubated with horseradish peroxidase-conjugated goat anti-mouse immunoglobulin for 30 minutes at room temperature. The signal was detected using a ready-to-use DAKO EnVision™ system (Dako, Glostrup, Denmark). The sections were subsequently counterstained with haematoxylin (Vector Laboratories, Burlingame, CA, USA) and viewed at five different fields per section using light microscopy. For the negative isotype controls, the primary antibody was replaced with a purified mouse immunoglobulin (1:500 dilution, Zymed-Invitrogen, Carlsbad, CA, USA). The images were captured with a Nikon epifluorescent upright microscope E600 (Nikon, Tokyo, Japan).

### SYBR Green I quantitative PCR (Q-PCR)

To validate the gene expression stability of the two potential reference marker candidates (*ACTB, HSP60*), real-time monitoring of the PCR reactions was performed according to standard procedures as described previously [10]. Total RNA was extracted from tissue samples by TRIzol reagent (Invitrogen, Carlsbad, CA, USA), followed by treatment with DNase I (Ambion, Austin, TX), and subsequently the amount and quality were determined by 2100 Bioanalyzer (Agilent Technologies, Inc., Santa Clara, CA, USA). Approximately 250 ng of DNase-treated RNA was used for RT reaction. The oligonucleotide primers for *ACTB *and *HSP60 *were designed using the ABI PRISM Primer Express Software (Applied Biosystems) (see Additional file 1).*HMBS *was used as an internal control, seeing that a recent publication from Cicinnati's group suggested that *HMBS *was found to be the single best reference gene for gene expression studies in HCC [12]. The fluorescence dyes, SYBR Green I was used as signal reporters to benefit a gain in sensitivity for signal detection.

### Q-PCR with fluorescent resonance energy transfer (FRET) hydrolysis probes

To test the gene expression stability of the two markers, Q-PCR was performed using FRET hydrolysis probes in order to gain in specificity for subsequently analysis. Primer and probe sequences were designed for the six most commonly used reference genes obtained from published literature: beta-2-microglobulin (*B2M*), hypoxanthine phosphoribosyl-transferase I (*HRPT1*), ribosomal protein L32 (*RPL32*), succinate dehydrogenase complex, subunit (*SDHA*), hydroxymethyl-bilane synthase (*HMBS*), ribosomal protein L13a (*RPL13A*); and the two reference markers identified in this study: beta-actin (*ACTB*) and heat shock protein 60 (*HSP60*) using the Universal Probe Library Assay Design Centre http://www.roche-applied-science.com (see Additional file 2). The accession numbers, functions, and chromosomal locations of these genes were also tabulated (see Additional file 3). The expression level of these 8 reference genes was assessed in 40 human liver samples (10 advanced stage HCC, 10 early stage HCC, 10 cirrhosis, and 10 normal liver tissues). The real-time assays were performed in triplicate using the Roche LightCycler 480 (Roche Diagnostic Ltd, UK). Thermal cycling was operated in the fast mode and the cycling parameter was as follows: 40 cycles of 95°C for 10 seconds and 60°C for 30 seconds. Real-time data was analysed using the Roche LightCycler 480 software (Roche Diagnostic Ltd).

### Statistical analysis

The 2D gel spot intensities were statistically analysed by SPSS Statistical Package version 13.0 for Window (SPSS, Chicago, IL) to perform one-way Analysis of Variance (ANOVA). P value (> 0.99) was employed purely as a ranking tool to identify proteins with potentially low variability across different diagnostic groups and does not imply any measure of statistical significance.

The stability of the reference transcripts was calculated using the geNorm algorithm described by Vandesompele *et al*. [13]. The C_T _value for each reference transcript was converted into a relative quantity, which was subsequently used to calculate the gene stability value (M). In addition, the pair-wise variation value (V) was analysed between candidate reference transcripts. This value acts as a parameter for denoting the optimum number of reference transcripts to be used when the expression level of certain transcripts are assayed in a specific cohort of clinical specimens.

## Results

### Beta-actin, HSP60, and PDI as putative reference markers in human liver tissues

**Figure 1:**
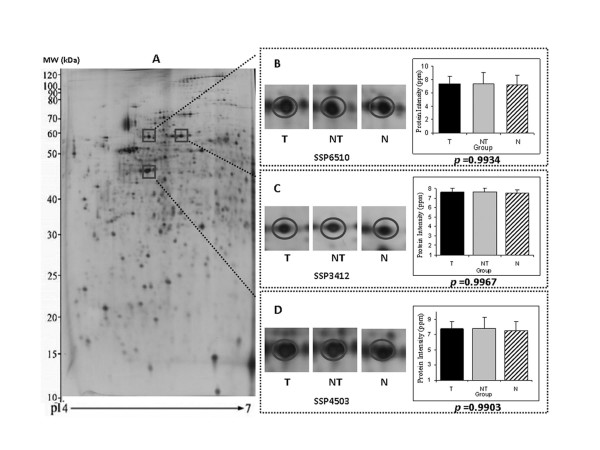
**Identification of beta-actin, HSP60, and PDI as potential housekeeping markers**. The protein expression profile of liver tissue on a 2-DE gel is shown, and the zones with the protein markers, beta-actin (SSP3412), HSP 60 (SSP4503), and PDI (SSP6510), are square-bracketed. Representative gel images and the histograms illustrating the relative protein intensities (in ppm) of SSP3412, SSP4503 and SSP6510 among tumourous, T (n = 105), non-tumourous cirrhotic, NT (n = 103), and normal, N (n = 16) liver tissues are shown. Statistical analysis was performed by one-way ANOVA and p value was used as a ranking system for expression variability. Data are presented as the mean ± SD.

In this study, the 2-DE expression profiles of tumourous, non-tumourous cirrhotic and normal liver proteomes are depicted (Figure 1). We found 1,433 proteins in human liver tissues on the 2-DE gel. After gel-to-gel matching and normalization, one-way ANOVA was performed and p-value analysis was used as a ranking tool to identify proteins with potentially low variability across different analysed groups. Three protein spots, labelled as SSP3412, SSP4503, and SSP6510, were selected based on their mean, standard deviation, 95% confidence interval and the calculated p values, suggested that these proteins exhibited a high rank with low expression variation across the tumourous, non-tumourous cirrhotic, and normal liver tissues (0.997, 0.990 and 0.993, respectively).

Often the mean value is quoted along with the standard deviation to describe the population distribution, where the mean summarises the central location of the data and the standard deviation shows the spread. Table 1 shows the mean and standard deviation of each reference protein marker candidate, mean intensities in ppm were more or less 7 in the three diagnostic groups with a low standard deviation indicated little variability of the data distribution. Moreover, 95% confidence interval also suggested that the population to possess a similar spread. To further justified the potential use of the three selected reference markers, overall measure of the variability was performed based on their mean, standard deviation and 95% CI of the combined groups (cancerous, non-tumourous cirrhotic, and normal). Results unambiguously verified the reliability of the choice of selection.

**Table 1:** Protein expression of SSP3412, SSP4503, and SSP6510 in tumourous, non-tumourous cirrhotic and normal liver tissues

Spot ID	Spot identity	Phenotypes	Mean Intensity (ppm)	95% CI	ANOVA (p-value*)
SSP3412	Beta-actin	Tumour	7.59 ± 0.89	5.82-9.37	**0.99**
		Non-tumour	7.61 ± 1.76	4.10-11.12	
		Normal	7.49 ± 1.02	5.45-9.54	
		Combined Groups	7.56 ± 1.22	5.12-10.00	-
					
SSP4503	Heat shock protein 60	Tumour	7.74 ± 0.94	5.85-9.62	**0.99**
		Non-tumour	7.73 ± 1.55	4.62-10.84	
		Normal	7.55 ± 1.12	5.30-9.79	
		Combined Groups	7.67 ± 1.20	5.27-10.07	-
					
SSP6510	Protein disulphide isomerase	Tumour	7.36 ± 1.08	5.20-9.52	**0.99**
		Non-tumour	7.39 ± 1.65	4.09-10.68	
		Normal	7.20 ± 1.43	4.35-10.05	
		Combined Groups	7.32 ± 1.39	4.54-10.10	-

* P-value does not imply any measure of statistical significance; it is apply as a ranking tool to identify proteins with low variability across different diagnostic groups.

Thus, these three protein spots were regarded as potential housekeeping candidates for application as internal protein reference controls. Using tandem MS, these three proteins were identified as beta-actin, HSP60, and PDI (see Additional file 4).

### Verification of beta-actin and HSP60 as stable genomic and proteomic reference markers

**Figure 2:**
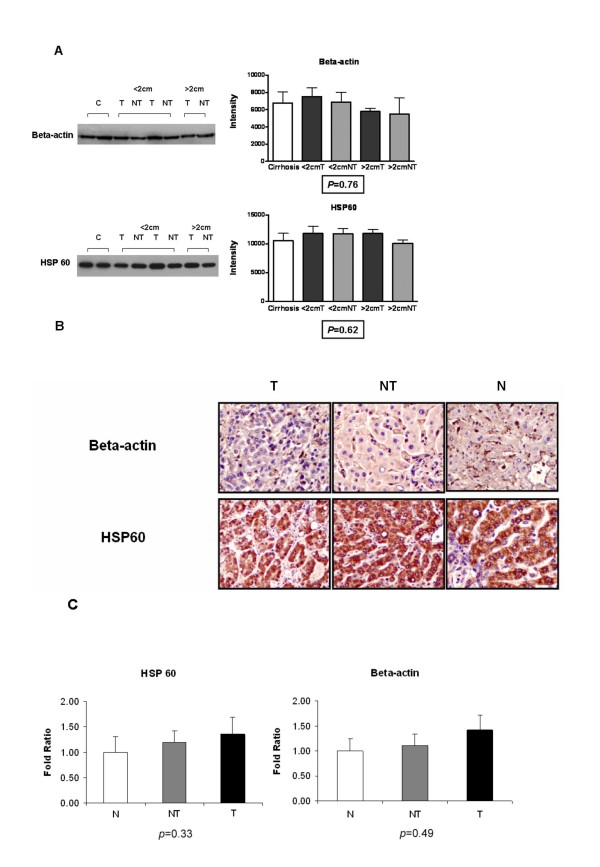
**Stability validation of potential housekeeping markers in protein and gene levels**. **(A) **Western blot demonstrated the expression stability of beta-actin and HSP 60. An equal amount of 25 μg of protein lysate was loaded per lane in order to measure the expression stability of beta-actin and HSP 60 among liver tissues with different conditions. Representative blot images were showed parallel to the histograms with protein intensities analysed in different liver tissues groups. C, Cirrhosis; < 2 cm, small size [less than (<) 2 cm] paired tumour (T) and non-tumour (NT); > 2 cm, large size [greater than (>) 2 cm] paired tumour (T) and non-tumour (NT). Statistical analysis was performed by one-way ANOVA. Data are presented as the mean ± SD. **(B) **Immunohistochemical staining showed the localizations and the levels of expression of beta-actin and HSP60 in tumourous, T (n = 30); non-tumourous cirrhotic, NT (n = 30); and normal, N (n = 16) liver tissues. Representative photos are shown (magnification: ×400). **(C) **Real-time qPCR was performed to evaluate the mRNA levels of beta-actin and HSP60 in tumourous, T (n = 20); non-tumourous cirrhotic, NT (n = 20); and normal, N (n = 16) liver tissues. Statistical analysis was performed by one-way ANOVA (*p *> 0.05). Data are presented as the mean ± SD.

Beta-actin belongs to the actin family and is the basic component of actin microfilaments [14]. HSP60 is a family member of the heat shock protein family, which includes the HSP's and glucose regulated proteins (GRP's) [15]. PDI performs chaperone functions in the endoplasmic reticulum by catalysing disulphide bond formation and isomerisation [16]. Only HSP 60 and beta-actin were successfully confirmed for their constant expression levels in both protein and transcript by western blot and qPCR respectively and therefore we excluded PDI as a potential candidate reference marker (see Additional file 5). *PDIA3 (protein disulfide isomerase family A, member 3) *is the gene encoded for PDI. The two verified proteins (HSP 60 and beta-actin) belong to distinctly different and unrelated protein families, by 2D gel analysis their expression levels were found to be stable in human liver tissues possessing various stages of HCC and these results were further confirmed using western blotting, (Figure. 2A), immunohistochemistry (Figure. 2B) and Q-PCR (Figure. 2C). The fold ratio difference across sample types shown in Figure 2C is small, and under 0.5 for both beta-actin and HSP60, which is in agreement with geNorm, and may well have no biological importance. No significant difference in their abundance and localizations were found among various samples. More importantly, expression of these reference markers was not associated with any of the clinicopathological parameters in the HCC tumour tissues (Table 2) and non-tumourous liver tissues (Table 3).

**Table 2:** The expressions of beta-actin and HSP60 in different clinicopathological subgroups of HCC tumour tissues.

Clinicopathological parameters	n = 105	Beta-actin		HSP60	
		
		Mean intensity (ppm)	*p *value	Mean intensity (ppm)	*p *value
**Gender**					
Male	89	7.60 ± 0.95	0.81^*a*^	7.77 ± 1.06	0.63^*a*^
Female	16	7.59 ± 0.93		7.70 ± 0.93	
**Age (years)**					
≥60	38	7.61 ± 0.81	0.76^*a*^	7.48 ± 0.74	0.67^*a*^
< 60	67	7.58 ± 1.01		7.76 ± 1.17	
**Tumour size (cm)**					
> 2	69	7.79 ± 0.96	0.17^*a*^	7.68 ± 0.98	0.91^*a*^
≤2	36	7.40 ± 0.89		7.79 ± 1.14	
**Venous infiltration**					
Yes	58	7.53 ± 0.71	0.61^*a*^	7.58 ± 0.73	0.36^*a*^
No	57	7.65 ± 1.01		7.89 ± 0.87	
**New TNM stage**					
Stage I-II	69	7.72 ± 1.10	0.38^*a*^	7.58 ± 0.99	0.75^*a*^
Stage III-IV	36	7.47 ± 0.84		7.70 ± 1.14	
**New Edmonson Grade**					
Well differentiated	27	7.49 ± 1.37	0.39^*b*^	7.68 ± 1.37	0.97^*b*^
Moderately differentiated	60	7.51 ± 0.94		7.76 ± 0.77	
Poorly differentiated	18	7.79 ± 0.71		7.77 ± 0.73	

*a*, analysed by independent *t *test; *b*, analysed by one-way ANOVA.

**Table 3:** The expressions of beta-actin and HSP60 in different clinicopathological subgroups of the non-tumourous liver tissues.

Clinicopathological parameters	n = 103	Beta-actin		HSP60	
		
		Mean intensity (ppm)	*p *value	Mean intensity (ppm)	*p *value
**Gender**					
Male	88	7.59 ± 1.93	0.77^*a*^	7.76 ± 1.76	0.55^*a*^
Female	15	7.63 ± 1.11		7.70 ± 0.79	
**Age (years)**					
≥60	34	7.82 ± 1.26	0.50^*a*^	7.89 ± 1.07	0.41^*a*^
< 60	69	7.40 ± 2.09		7.57 ± 1.91	
**HBsAg**					
Positive	89	7.70 ± 1.77	0.82^*a*^	7.87 ± 1.76	0.49^*a*^
Negative	14	7.52 ± 2.17		7.59 ± 0.92	
**Liver histology**					
Chronic active hepatitis B	34	7.94 ± 1.75	0.91^*b*^	7.45 ± 0.85	0.34^*b*^
Mildly cirrhotic	44	7.50 ± 1.01		7.72 ± 1.17	
Moderately cirrhotic	22	7.69 ± 0.66		7.50 ± 0.50	
Severely cirrhotic	3	7.31 ± 0.46		8.25 ± 0.42	

*a*, analysed by independent *t *test; *b*, analysed by one-way ANOVA.

### Gene-stability measure and pairwise variation analysis

**Figure 3:**
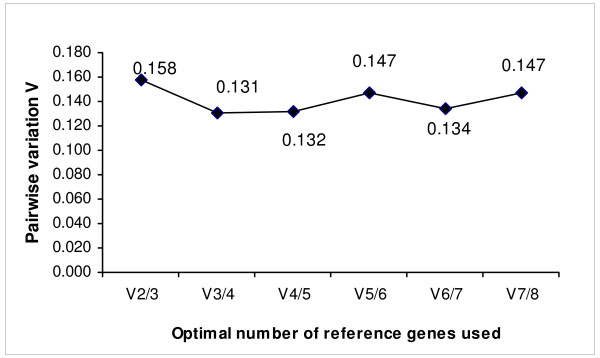
**The pairwise variation (V_n/n+1_) analysis**. The relative expressions of the 8 reference genes were assayed using Q-PCR and the pair-wise variation values (V) were determined using geNorm algorithm in order to find the optimum number of reference genes required for accurate data normalization.

Next, we employed the mathematical model (geNorm algorithm) to investigate the gene expression stability of *ACTB*, and *HSP60*, also to determine the optimal number of reference genes that are required to perform an accurate normalisation in a defined set of experimental specimens [13]. The threshold cycle values (C_T _values) of the eight reference genes [beta-2-microglobulin (*B2M*), hypoxanthine phosphoribosyl-transferase I (*HRPT1*), ribosomal protein L32 (*RPL32*), succinate dehydrogenase complex, subunit (*SDHA*), hydroxymethyl-bilane synthase (*HMBS*), ribosomal protein L13a (*RPL13A*), beta-actin (*ACTB*) and heat shock protein 60 (*HSP60*)] in each sample group were averaged and used to determine the relative quantities, and based on that, the gene stability (M) and pair-wise variation (V) values of the reference gene in the set of samples were calculated (see Additional file 6 and Table 4). Table 4 showed the priority ranking of the eight reference genes according to their gene stabilities, which were analysed by geNorm software in the combined set of liver tissue samples. No other usual analytical software (such as NormFinder) was employed because previous experience had shown the different softwares usually derive similar results as GeNorm. *ACTB *and *HSP 60 *exhibited the lowest M value in all liver tissue groups (normal, cirrhosis and HCC) suggested that the finding was in agreement with the protein data derived from the 2-DE profiling method as shown above, in which the proteins encoded by *ACTB *and *HSP60 *were found to have stable expression in human liver tissues. Akin to the original publication of geNorm, a pair-wise variation value of 0.15 was used as a cut-off point that indicates the optimum number of genes used for normalization in a gene expression study with high confidence [13]. This study showed that two reference genes provided a geNorm score of 0.158 which was very close to the 0.15 geNorm cut-off value. Therefore, in subsequent studies we recommend that two reference genes would be sufficient to be employed for normalising gene expression in human hepatic tissues (Figure 3 and Table 4).

**Table 4:** The ranking of reference gene expression stability in set of human liver tissues

Ranking order*	HCC, Cirrhosis and Normal	M	V
8	HPRT1	0.94	0.147
7	SDHA	0.82	0.134
6	B2M	0.74	0.147
5	RPL13A	0.63	0.132
4	HMBS	0.55	0.131
3	RPL32	0.51	**0.158**

**The 2 most stably expressed genes**	**ACTB, HSP60**	**0.47**	

Gene stability increased from top to bottom; M, gene stability value; V, pairwise stability value

## Discussion

Proteome profiling of different stages of hepatocarcinogenesis has been well studied [9] and this has resulted in the identification of a panel of differentially expressed proteins at each stage. However, proteins with constant expression during the oncogenesis of HCC have not yet been characterized. To be eligible as a housekeeping protein, the level of the expression of the protein should not change during cell development, in response to drug treatment, or in disease. Estimation of relative expression of genes or proteins are often made by comparison against the internal standards which for transcriptomics using DNA arrays or PCR, or for proteins when using western blotting or protein arrays. For instance, western blotting is often used for exploration and confirmation of many proteins with normalisation against "housekeeping proteins" to correct for protein loading and factors, such as transfer efficiency [8]. Hypothetical regulation of a housekeeping protein, e.g. between different disease status or between different tissue types, could potentially lead to the conclusion that the target protein was regulated, but actually this was due to dysregulation of the housekeeping protein rather than the target protein itself. This renders the identification of housekeeping proteins highly significant in expressional studies in clinical research.

Although the specific quantities and forms of the protein produced are highly regulated during complex cellular translational and post-translational processes, a poor correlation between mRNA and protein abundance is often documented [17]. Given this caveat, the aim of the study was to investigate whether protein(s) patterns of expression are constant in oncogenesis of liver cancer. These markers were referred to as potential housekeeping proteins, which can be isolated and identified using a proteomic platform and hereafter correlated with their mRNA abundance.

In the present study, we have employed the 2-DE-based proteomic approach to investigate 224 liver proteomes which represented various clinical conditions. We have successfully identified certain reference protein markers that were evenly expressed in liver tissues among the healthy, cirrhotic and malignant states by employing p-value as a ranking system; and comparing the variability using the mean, standard deviation and 95% CI. Methodology was kept in the linear range from sample preparation, to gel silver staining. In addition, the background of each gel was compared by two experienced operators independently to assess the similarity of protein spots and normalised using PDQuest (Bio-Rad) before proceeding to analysis. The three most stable markers were characterized by MALDI-TOF/MS as beta-actin (ACTB), heat shock protein 60 (HSP60), and protein disulphide isomerase (PDI), and the corresponding expression patterns were further determined by western blot, immunohistochemistry and Q-PCR. However, only ACTB and HSP 60 had been successfully fulfilled the complete validation process which included transcript expression. Herein, we postulate these two markers to be active participants in the normal physiological functions and do not possess essential roles in liver cirrhosis and hepatocarcinogenesis. However, some other studies demonstrated otherwise [18,19], which may reflect on the precise nature of sample types being analysed, in that optimum selection of reference genes depends on the specific cohort of samples, the method used to retrieve these samples and sample preparation and treatments.

For instance, the expression of beta-actin was found up-regulated in liver tumour samples when compared with normal liver samples, implying a correlation between over-expression of beta-actin and oncogenesis in hepatoma [20]. On the other hand, HSP60, belongs to the HSP family and the members of this family are frequently dysregulated in malignant conditions [21]. For instance, HSP60 was over-expressed in colorectal [22] and cervical [23] cancers, but down-regulated in urinary bladder cancer [21]. However, these observations are controversial and are contrary to our findings that have been performed in a larger cohort study. Indeed, beta-actin has been widely used as an internal reference gene/protein in real-time PCR and western blotting in liver tissues or others. Our data also revealed that the proposed reference markers (ACTB and HSP60) were expressed equivalently in diseased or healthy tissues by 2-DE profiling, western blot, IHC, Q-PCR and mathematical modelling. In fact, *HMBS *was initially used as an internal control for validation of the two potential reference marker candidates mainly because *HMBS *was previously suggested to be the single best reference gene for gene expression studies in HCC [12]. The reference gene was subsequently included for evaluation together with other conventional reference genes and the two potential reference candidates identified from our study. *ACTB *and *HSP 60 *were identified to be superior to *HMBS *as references candidates for HCC gene expression studies (Table 4). Therefore, it is believed that these proteins are key components that are involved in basic cellular structure and functions regardless of the pathophysiological states.

The discovery that protein abundance of ACTB and HSP60 across diseased and healthy tissues are similarly analogous is important because imprecise sampling between diseased and healthy tissue, and the inherently heterogeneous nature of liver tissues is not a point for conjecture when measuring dysregulation of disease associated proteins relative to these very stably expressed housekeeping proteins. Protein levels were confirmed using 2-DE, immunoblotting and immunohistochemistry. These methods are not renowned for their ability to quantify proteins, while methodological approaches improve [8], this study has drawn on the quantitative power offered by two qPCR procedures. Moreover, the present study has been careful to use ANOVA analysis of 2-DE gels and has gone further to show a close positive correlation between protein and mRNA abundance for the reference candidates ACTB and HSP60. The detailed application of protein and mRNA extraction and analysis as shown in this study may provide for a generic means to better correlate transcript level to protein production, and could possibly lead to the prediction of dys-regulated proteins related to pathogenesis from mRNA screens.

## Conclusion

The use of reference molecules with stable expression across tissue sample cohorts is important in clinical research, particularly when the expression levels of certain molecules are compared and used to predict pathological conditions [24]. Our results demonstrated the high expression stability of *ACTB *and *HSP60 *in human liver tissues both in protein and transcript levels and their positive correlation in human liver samples. Moreover, using geNorm algorithm showed at least 2 reference markers were recommended for data normalization. A prospective, multicenter study is under way for validation of this protein-based set of reference markers in high-risk population for HCC.

## Abbreviations

2-DE: two-dimensional gel electrophoresis; ACTB: beta-actin; HCC: hepatocellular carcinoma; N: healthy donor; HSP60: heat shock protein 60; IHC: immunohistochemistry; NT: non-tumour; T: tumour; C: cirrhosis.

## Competing interests

The authors declare that they have no competing interests.

## Authors' contributions

SS and XY performed all experiments, data analysis and interpretation. JML and PJD contributed to the study design and conception. CY help in the acquisition of clinical samples and gave advices on samples' clinical background. JML, PJD and RTP carried out critical revision of the manuscript. The manuscript was drafted and written by SS and approved by all the authors.

## Pre-publication history

The pre-publication history for this paper can be accessed here:

http://www.biomedcentral.com/1471-2407/9/309/prepub

## Supplementary Material

Additional file 1 **Primer sequences for beta-actin, heat shock protein 60 and HMBS used in SYBR Green I quantitative PCR**. Detail of the primer sequences such as Tm, primer length and amplicon size are provided for the quantitative PCR study. Click here for file

Additional file 2 **Primer and probe sequences of 8 reference genes used in Q-PCR with FRET hydrolysis probes**. Detail of the forward and reverse primer sequences and universal probe number are summarized in the table. Click here for file

Additional file 3 **8 reference genes evaluated in this study**. Description of the function and chromosomal localization of the 8 reference genes evaluated in the study. Click here for file

Additional file 4 **Mass spectrometry results for SSP3412, SSP4503, and SSP6510**. The data provided such as protein mass, score and queries matched explicitly confirmed the identity of the proteins. Click here for file

Additional file 5 **The expression levels of PDI/*PDIA3 *in human hepatic tissues of different liver diagnostic groups**. A) by western blot analysis and B) by real-time quantitative PCR. The protein levels of PDI show a discrepancy across different hepatic liver tissues (1- Cirrhosis; 2- < 2 cm NT; 3- < 2 cm T; 4- > 2 cm NT; 5- > 2 cm T) using 25 μg of protein per lane, although its transcript levels are fairly stable. As a result, PDI was excluded as a potential candidate reference marker. Data are presented as the mean ± SD. Click here for file

Additional file 6 **The expression levels of 8 internal reference genes in four groups of human liver tissues**. Q-PCR was performed for the 8 reference genes and their mean C_T _values ( ± SD) of four groups of human liver tissues (advanced stage HCC, early stage HCC, cirrhosis, and normal liver) were presented in a histogram. Click here for file

## References

[B1] Sun S Lee NP Poon RT Fan ST He QY Lau GK Luk JM Oncoproteomics of hepatocellular carcinoma: from cancer markers' discovery to functional pathways Liver Int 2007 27 8 1021 1038 10.1111/j.1478-3231.2007.01533.x 17845530

[B2] Lee NP Cheung ST Poon RT Fan ST Luk JM Genomic and proteomic biomarkers for diagnosis and prognosis of hepatocellular carcinoma Biomarkers Med 2007 1 2 273 284 10.2217/17520363.1.2.273 20477402

[B3] Lee NP Leung KW Cheung N Lam BY Xu MZ Sham PC Lau GK Poon RT Fan ST Luk JM Comparative proteomic analysis of mouse livers from embryo to adult reveals an association with progression of hepatocellular carcinoma Proteomics 2008 8 10 2136 2149 10.1002/pmic.200700590 18425728

[B4] de Kok JB Roelofs RW Giesendorf BA Pennings JL Waas ET Feuth T Swinkels DW Span PN Normalization of gene expression measurements in tumor tissues: comparison of 13 endogenous control genes Lab Invest 2005 85 1 154 159 15543203 10.1038/labinvest.3700208

[B5] Fu LY Jia HL Dong QZ Wu JC Zhao Y Zhou HJ Ren N Ye QH Qin LX Suitable reference genes for real-time PCR in human HBV-related hepatocellular carcinoma with different clinical prognoses BMC Cancer 2009 9 49 10.1186/1471-2407-9-49 19200351 PMC2644316

[B6] Winer J Jung CK Shackel I Williams PM Development and validation of real-time quantitative reverse transcriptase-polymerase chain reaction for monitoring gene expression in cardiac myocytes in vitro Anal Biochem 1999 270 1 41 49 10.1006/abio.1999.4085 10328763

[B7] Cappelli K Felicetti M Capomaccio S Spinsanti G Silvestrelli M Supplizi AV Exercise induced stress in horses: selection of the most stable reference genes for quantitative RT-PCR normalization BMC Mol Biol 2008 9 49 10.1186/1471-2199-9-49 18489742 PMC2412902

[B8] Ferguson RE Carroll HP Harris A Maher ER Selby PJ Banks RE Housekeeping proteins: a preliminary study illustrating some limitations as useful references in protein expression studies Proteomics 2005 5 2 566 571 10.1002/pmic.200400941 15627964

[B9] Luk JM Lam CT Siu AF Lam BY Ng IO Hu MY Che CM Fan ST Proteomic profiling of hepatocellular carcinoma in Chinese cohort reveals heat-shock proteins (Hsp27, Hsp70, GRP78) up-regulation and their associated prognostic values Proteomics 2006 6 3 1049 1057 10.1002/pmic.200500306 16400691

[B10] Yi X Luk JM Lee NP Peng J Leng X Guan XY Lau GK Beretta L Fan ST Association of mortalin (HSPA9) with liver cancer metastasis and prediction for early tumor recurrence Mol Cell Proteomics 2008 7 2 315 325 17934217 10.1074/mcp.M700116-MCP200

[B11] Wong BW Luk JM Ng IO Hu MY Liu KD Fan ST Identification of liver-intestine cadherin in hepatocellular carcinoma--a potential disease marker Biochem Biophys Res Commun 2003 311 3 618 624 10.1016/j.bbrc.2003.10.032 14623315

[B12] Cicinnati VR Shen Q Sotiropoulos GC Radtke A Gerken G Beckebaum S Validation of putative reference genes for gene expression studies in human hepatocellular carcinoma using real-time quantitative RT-PCR BMC Cancer 2008 8 350 10.1186/1471-2407-8-350 19036168 PMC2607287

[B13] Vandesompele J De Preter K Pattyn F Poppe B Van Roy N De Paepe A Speleman F Accurate normalization of real-time quantitative RT-PCR data by geometric averaging of multiple internal control genes Genome Biol 2002 3 7 R0034.0031 R0034.0011 10.1186/gb-2002-3-7-research0034 PMC126239 12184808

[B14] Khaitlina SY Functional specificity of actin isoforms Int Rev Cytol 2001 202 35 98 full_text 11061563 10.1016/s0074-7696(01)02003-4

[B15] Garrido C Gurbuxani S Ravagnan L Kroemer G Heat shock proteins: endogenous modulators of apoptotic cell death Biochem Biophys Res Commun 2001 286 3 433 442 10.1006/bbrc.2001.5427 11511077

[B16] Klappa P Hawkins HC Freedman RB Interactions between protein disulphide isomerase and peptides Eur J Biochem 1997 248 1 37 42 10.1111/j.1432-1033.1997.t01-1-00037.x 9310357

[B17] Gygi SP Rochon Y Franza BR Aebersold R Correlation between protein and mRNA abundance in yeast Mol Cell Biol 1999 19 3 1720 1730 10022859 10.1128/mcb.19.3.1720 PMC83965

[B18] Gao Q Wang XY Fan J Qiu SJ Zhou J Shi YH Xiao YS Xu Y Huang XW Sun J Selection of reference genes for real-time PCR in human hepatocellular carcinoma tissues J Cancer Res Clin Oncol 2008 134 9 979 986 10.1007/s00432-008-0369-3 18317805 PMC12160763

[B19] Waxman S Wurmbach E De-regulation of common housekeeping genes in hepatocellular carcinoma BMC Genomics 2007 8 243 10.1186/1471-2164-8-243 17640361 PMC1937003

[B20] Chang TJ Juan CC Yin PH Chi CW Tsay HJ Up-regulation of beta-actin, cyclophilin and GAPDH in N1S1 rat hepatoma Oncol Rep 1998 5 2 469 471 9468581 10.3892/or.5.2.469

[B21] Lebret T Watson RW Molinie V O'Neill A Gabriel C Fitzpatrick JM Botto H Heat shock proteins HSP27, HSP60, HSP70, and HSP90: expression in bladder carcinoma Cancer 2003 98 5 970 977 10.1002/cncr.11594 12942564

[B22] Cappello F Bellafiore M Palma A David S Marciano V Bartolotta T Sciume C Modica G Farina F Zummo G 60 KDa chaperonin (HSP60) is over-expressed during colorectal carcinogenesis Eur J Histochem 2003 47 2 105 110 12777205 10.4081/814

[B23] Cappello F Bellafiore M Palma A Marciano V Martorana G Belfiore P Martorana A Farina F Zummo G Bucchieri F Expression of 60-kD heat shock protein increases during carcinogenesis in the uterine exocervix Pathobiology 2002 70 2 83 88 10.1159/000067304 12476033

[B24] Giricz O Lauer-Fields JL Fields GB The normalization of gene expression data in melanoma: investigating the use of glyceraldehyde 3-phosphate dehydrogenase and 18S ribosomal RNA as internal reference genes for quantitative real-time PCR Anal Biochem 2008 380 1 137 139 10.1016/j.ab.2008.05.024 18554498 PMC2515093

