# Supplementary material for: A protein-based set of reference markers for liver tissues and hepatocellular carcinoma
Source: BMC Cancer. 2009 Sep 2;9:309. doi: 10.1186/1471-2407-9-309 (PMC2742551; doi:10.1186/1471-2407-9-309)
Supplement: Additional file 1 — Primer sequences for beta-actin, heat shock protein 60 and HMBS used in SYBR Green I quantitative PCR. Detail of the primer sequences such as Tm, primer length and amplicon size are provided for the quantitative PCR study. [file 1471-2407-9-309-S1.doc]

| **Additional file 1. Primer sequences for beta-actin, heat shock protein 60 and HMBS used in SYBR Green I quantitative PCR.** | | | | |
| --- | --- | --- | --- | --- |
| **Genes** | **Sequences (5' -> 3')** | **Tm (°C)** | **Primer Length** | **Amplicon Size (bp)** |
| Beta-actin | Forward: CATGTACGTTGCTATCCAGGC | 60.8 | 21 | 250 |
|  | Reverse: CTCCTTAATGTCACGCACGAT | 60.2 | 21 |  |
| Heat shock protein 60 | Forward: CTTCGGTTACCCACAGTCTTTC | 60.6 | 22 | 126 |
|  | Reverse: ACCTTGAAGCATTAAGGCTCG | 60.4 | 21 |  |
| **Internal control** | **Sequence (5' -> 3')** | | | |
| Hydroxymethyl-bilane synthase (*HMBS*) | Forward: AGCTATGAAGGATGGGCAAC | | | |
|  |  |  |  |  |
|  | Reverse: TTGTATGCTATCTGAGCCGTCTA | | | |
