# Supplementary material for: A protein-based set of reference markers for liver tissues and hepatocellular carcinoma
Source: BMC Cancer. 2009 Sep 2;9:309. doi: 10.1186/1471-2407-9-309 (PMC2742551; doi:10.1186/1471-2407-9-309)

**Additional file 2. Primer and probe sequences of 8 reference genes used in Q-PCR with FRET hydrolysis probes.**


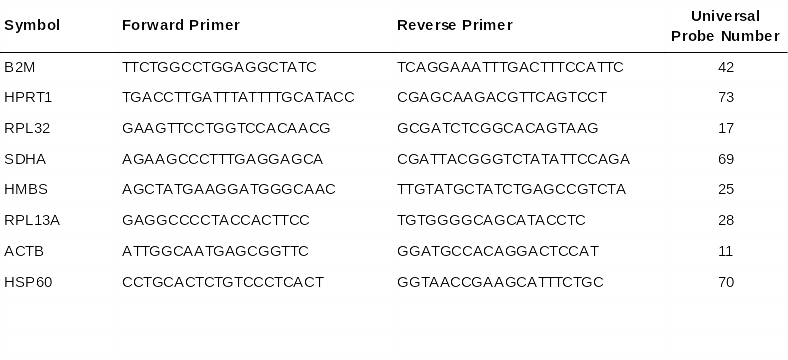

Supplement: Additional file 2 — Primer and probe sequences of 8 reference genes used in Q-PCR with FRET hydrolysis probes. Detail of the forward and reverse primer sequences and universal probe number are summarized in the table. [file 1471-2407-9-309-S2.doc]
