# Supplementary material for: A protein-based set of reference markers for liver tissues and hepatocellular carcinoma
Source: BMC Cancer. 2009 Sep 2;9:309. doi: 10.1186/1471-2407-9-309 (PMC2742551; doi:10.1186/1471-2407-9-309)
Supplement: Additional file 3 — 8 reference genes evaluated in this study. Description of the function and chromosomal localization of the 8 reference genes evaluated in the study. [file 1471-2407-9-309-S3.doc]

**Additional file 3. 8 reference genes evaluated in this study.**

| **Symbol** | **Accession number** | **Name** | **Function** | **Chromosomal localization** |
| --- | --- | --- | --- | --- |
| *B2M* | NM_004048 | Beta-2-microglobulin | Beta-chain of major histocompatibility complex class I molecules | 15q21-q22 |
| *HRPT1* | NM_000194 | Hypoxanthine phosphoribosyl-transferase I | Prine synthesis in salvage pathway | Xq26 |
| *RPL32* | NM_000994 | Ribosomal protein L32 | Structural component of the 60S subunit, belongs to L32E family of ribosomal protein | 3p25-p24 |
| *SDHA* | NM_004168 | Succinate dehydrogenase complex, subunit | An electron transporter in the TCA cycle respiratory chain | 5p15 |
| *HMBS* | NM_000190 | Hydroxymethyl-bilane synthase | Heme synthesis, porphyrin metabolism | 11q23 |
| *RPL13A* | NM_012423 | Ribosomal protein L13a | Structural component of the large 60S ribosomal subunit, belongs to L13P family of ribosomal proteins | 19q13 |
| *ACTB* | NM_001101 | Beta-actin | Cytoskeletal structural protein | 7p15-p12 |
| *HSP60* | NM_002156 | Heat shock protein 60 | Signaling molecule in the innate immune system | 2q33.1 |
