# Supplementary material for: A protein-based set of reference markers for liver tissues and hepatocellular carcinoma
Source: BMC Cancer. 2009 Sep 2;9:309. doi: 10.1186/1471-2407-9-309 (PMC2742551; doi:10.1186/1471-2407-9-309)
Supplement: Additional file 4 — Mass spectrometry results for SSP3412, SSP4503, and SSP6510. The data provided such as protein mass, score and queries matched explicitly confirmed the identity of the proteins. [file 1471-2407-9-309-S4.doc]

| **Additional file 4. Mass spectrometry results for SSP3412, SSP4503, and SSP6510.** | | | | | | | | | |
| --- | --- | --- | --- | --- | --- | --- | --- | --- | --- |
| **SSP3412: Beta-actin (gi:14250401)**  **Mass: 42128 Score: 120 Queries matched: 2** | | | | | | | | | |
| Query | **Observed** | **Mr**  **(expt)** | **Mr**  **(calc)** | **Delta** | **Miss** | **Score** | **Expect** | **Rank** | **Peptide(s)** |
| 1 | 976.5 | 975.5 | 975.5 | 0.05 | 0 | 40 | 0.0065 | 8 | **K.AGFAGDDAP** |
| 3 | 1132.5 | 1131.5 | 1131.5 | -0.03 | 0 | 80 | 0.0004 | 1 | **R.GYSFTTTAE** |
|  | | | | | | | | | |
| **SSP4503: HSP60 (gi:306890) Mass: 61157 Score: 100 Queries matched: 5** | | | | | | | | | |
| **Query** | **Observed** | **Mr**  **(expt)** | **Mr (calc)** | **Delta** | **Miss** | **Score** | **Expect** | **Rank** | **Peptide(s)** |
| 1 | 855.9 | 854.5 | 854.5 | 0.03 | 0 | 6 | 43 | 4 | **K.GANPVEIR** |
| 2 | 960.5 | 959.5 | 859.9 | -0.01 | 0 | 23 | 1.3 | 1 | **R.VTDALNATR** |
| 3 | 1684.9 | 1683.9 | 1683.9 | 0 | 0 | 26 | 0.32 | 1 | **R.AAVEEGIVL** |
| 4 | 1771.8 | 1770.8 | 1770.9 | -0.05 | 0 | 37 | 0.022 | 1 | **R.CIPALDSLT** |
| 5 | 1919.1 | 1918.1 | 1918.1 | 0.03 | 0 | 8 | 6.3 | 1 | **K.ISSIQSIVP** |
|  | | | | | | | | | |
| **SSP6510: PDI (gi:860986) Mass: 57043 Score: 123 Queries matched: 6** | | | | | | | | | |
| **Query** | **Observed** | **Mr**  **(expt)** | **Mr**  **(calc)** | **Delta** | **Miss** | **Score** | **Expect** | **Rank** | **Peptide(s)** |
| 1 | 995.6 | 994.6 | 994.6 | 0.04 | 0 | 9 | 15 | 1 | **K.QAGPASVPL** |
| 2 | 1172.5 | 1171.5 | 1171.5 | -0.04 | 0 | 12 | 6.1 | 1 | **K.FVMQEEFSR** |
| 3 | 1191.6 | 1190.6 | 1190.6 | 0 | 0 | 13 | 10 | 3 | **R.LAPEYEAAA** |
| 4 | 1359.6 | 1358.6 | 1358.7 | -0.06 | 0 | 9 | 13 | 1 | **R.FLQDYFDGN** |
| 5 | 1370.7 | 1369.7 | 1369.7 | 0 | 0 | 44 | 0.0066 | 1 | **R.ELSDFISYL** |
| 6 | 1664.8 | 1663.8 | 1663.8 | 0.04 | 0 | 37 | 0.03 | 1 | **K.MDATANDVF** |
