# Supplementary material for: A protein-based set of reference markers for liver tissues and hepatocellular carcinoma
Source: BMC Cancer. 2009 Sep 2;9:309. doi: 10.1186/1471-2407-9-309 (PMC2742551; doi:10.1186/1471-2407-9-309)
Supplement: Additional file 5 — The expression levels of PDI/PDIA3 in human hepatic tissues of different liver diagnostic groups. A) by western blot analysis and B) by real-time quantitative PCR. The protein levels of PDI show a discrepancy across different hepatic liver tissues (1- Cirrhosis; 2- < 2 cm NT; 3- < 2 cm T; 4- > 2 cm NT; 5- > 2 cm T) using 25 μg of protein per lane, although its transcript levels are fairly stable. As a result, PDI was excluded as a potential candidate reference marker. Data are presented as the mean ± SD. [file 1471-2407-9-309-S5.doc]

**
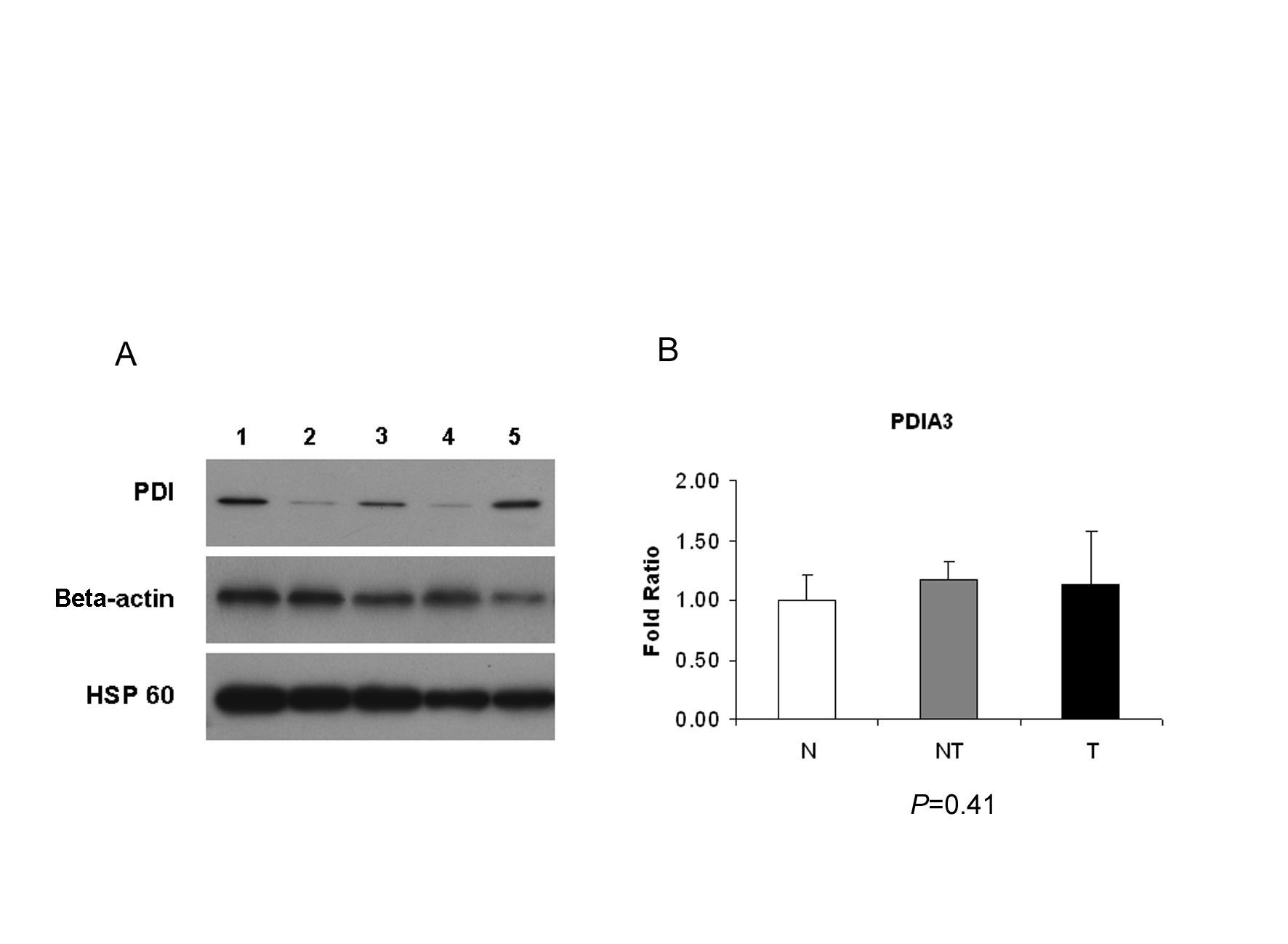
**

**Additional File 5: The expression levels of PDI/*PDIA3* in human hepatic tissues of different liver diagnostic groups.** A) by western blot analysis and B) by real-time quantitative PCR. The protein levels of PDI show a discrepancy across different hepatic liver tissues (1- Cirrhosis; 2- <2cm NT; 3- <2cm T; 4- >2cm NT; 5- >2cm T) using 25 μg of protein per lane, although its transcript levels are fairly stable. As a result, PDI was excluded as a potential candidate reference marker. Data are presented as the mean±SD.
