# Supplementary material for: A protein-based set of reference markers for liver tissues and hepatocellular carcinoma
Source: BMC Cancer. 2009 Sep 2;9:309. doi: 10.1186/1471-2407-9-309 (PMC2742551; doi:10.1186/1471-2407-9-309)
Supplement: Additional file 6 — The expression levels of 8 internal reference genes in four groups of human liver tissues. Q-PCR was performed for the 8 reference genes and their mean CT values ( ± SD) of four groups of human liver tissues (advanced stage HCC, early stage HCC, cirrhosis, and normal liver) were presented in a histogram. [file 1471-2407-9-309-S6.doc]

**Additional File 6: The expression levels of 8 internal reference genes in four groups of human liver tissues.** Q-PCR was performed for the 8 reference genes and their mean C_T_ values (± SD) of four groups of human liver tissues (advanced stage HCC, early stage HCC, cirrhosis, and normal liver) were presented in a histogram.
